# Implications of primary tumor site and fraction size on outcomes of palliative radiation for osseous metastases

**DOI:** 10.3389/fonc.2025.1432916

**Published:** 2025-03-31

**Authors:** Riley P. McDougall, Quoc-Anh Ho, Charles Hsu, Jared R. Robbins

**Affiliations:** ^1^ Department of Radiation Oncology, University of Arizona College of Medicine-Tucson, Tucson, AZ, United States; ^2^ Department of Radiation Oncology, Stanford University School of Medicine, Palo Alto, CA, United States; ^3^ Department of Radiation Oncology, Duke University School of Medicine, Durham, NC, United States

**Keywords:** PRLSRT, palliative radiation, NCDB (national cancer database), metastatic bone disease, palliative rt fractionation

## Abstract

**Purpose:**

This study reviewed palliative radiation therapy (RT) practices and outcomes and compared the percentage of remaining life spent receiving RT (PRLSRT) in patients treated for osseous metastases.

**Methods:**

A retrospective analysis was conducted using the National Cancer Database (2010–2016) to evaluate metastatic patients who received palliative bone RT. Common palliative RT schemes were analyzed to determine treatment patterns and outcomes. Palliative outcomes, including median PRLSRT, RT completion, and mortality rates, were calculated. Binary logistic regression was performed to identify factors affecting RT completion, and a scoring system was developed to identify patients at risk for poor palliative outcomes.

**Results:**

A total of 50,929 patients were included, with the majority diagnosed with NSCLC (45.2%), breast cancer (15.1%), or prostate cancer (10.8%). The median overall survival after palliative RT was 5.74 months. Patients receiving lower doses per fraction (2.5 Gy/Fx) tended to be younger, healthier, and yet experienced worse palliative outcomes. Binary logistic regression identified age, race, income quartile, and Gy/Fx as significant factors affecting RT completion. Median PRLSRTs were as follows: 14.95% for GI NOS, 9.89% for upper GI, 9.46% for NSCLC, 8.67% for skin, 7.06% for SCLC, 6.10% for lower GI, 5.59% for GYN, 5.44% for GU, 5.35% for HNC, 2.05% for endocrine, 2.03% for prostate cancer, and 1.82% for breast cancer. Patients receiving 2.5 and 3 Gy/Fx were less likely to complete RT compared to those receiving 4 Gy/Fx (OR, 1.429 and 3.780, respectively; *p* < 0.001). Age, comorbidities, primary tumor, target location, and metastatic burden were associated with PRLSRT ≥ 25%.

**Conclusion:**

Dose regimens and patient selection influence palliative bone RT outcomes. Both factors should be carefully considered to minimize the burden of care and maximize treatment benefits.

## Introduction

Bone metastasis is a prevalent complication of advanced cancer, with the skeletal system being the third most common site for metastases in patients with solid tumors (1). Solid tumor cancers that have spread to the bone can cause pain, neurological issues, and fractures, negatively impacting patients’ quality of life. Palliative radiotherapy (RT) is used to alleviate pain, prevent or reduce neurological dysfunction, and maintain bone stability (2). Various radiation doses have been studied, and fractionation schedules of 8 Gy/1 Fx, 20–24 Gy/5–6 Fx, 30 Gy/10 Fx, and 35–37.5 Gy/14–15 Fx are widely used to treat bone metastases (3–6). However, for patients with end-stage cancer, radiation dose fractionation and the number of treatments can significantly impact their quality of life. Additionally, physicians often underestimate the physical, financial, and logistical burden of treatment on terminally ill patients, while also overestimating their prognosis (7, 8). Therefore, assessing the impact of treatment schemas on palliative outcomes is crucial for improving patients’ quality of life (9, 10). This study aims to review current palliative RT practice patterns and evaluate the effectiveness of various treatment schemes.

## Materials and methods

Using the National Cancer Database, we evaluated patients with solid tumors and known osseous metastases who received RT to the bone between 2010 and 2016. Patients were selected based on radiation treatment volume codes (42, 80–86, 88, 98, 99) and common palliative RT dose-fractionation schemes (35–37.5 Gy/14–15 Fx, 30 Gy/10 Fx, 20–24 Gy/5–6 Fx, and 8 Gy/1 Fx). Exclusion criteria included patients receiving SBRT, those with unknown or missing RT doses, unknown or unspecified treatment volumes, dose-fractionation schemes indicative of potential primary treatment, and patients with hematologic or unknown primary cancers. Practice patterns were analyzed to identify common palliative RT schemes in relation to patient demographics, overall survival, and palliative outcomes, including RT incompletion rates and the percentage of remaining life spent receiving RT (PRLSRT).

Median survival was calculated from the start of RT and from the initiation of any treatment intervention. Cox proportional hazards analysis was conducted to identify factors correlated with overall survival from the start of any intervention. Mortality rates after starting RT were calculated and compared across dose-fractionation regimens to evaluate palliative outcomes. Patients who died within 2 days of starting RT were classified as having died during treatment. Radiation completion was assessed by comparing each dose per fraction with the anticipated total dose according to common fractionation schemes. Binary logistic regression was conducted to evaluate RT course completion based on significant patient characteristics and dose-per-fraction.

Additionally, we calculated the PRLSRT as follows:


$$\text{PRLSRT}=100\frac{\text{Elapsed days on RT}}{\text{Overall survival from the start of RT}}$$

To ensure data accuracy, reported elapsed days were compared with the number of fractions administered, with the mode of elapsed days used to resolve discrepancies. Median PRLSRT was calculated based on primary tumor locations and radiation schemes. Patients were stratified into four groups by PRLSRT for further demographic and treatment evaluation. Regimen incompletion rates and median PRLSRTs were also analyzed.

To identify variables contributing to worse PRLSRT outcomes, the total cohort PRLSRT was hypothesized and recalculated for all patients, assuming they had received the most common RT regimen (30 Gy/10 Fx). Patients with a death or last known contact within 12 days were assigned a PRLSRT of 100%. Binary logistic regression was performed on a randomly selected half of the cases to identify factors associated with a PRLSRT ≥ 25% under a 30-Gy/10-Fx regimen. A predictive score was established using the odds ratio of variables from the regression, and ROC analysis was conducted on the tested data before validation with the remaining unselected cases. The predictive scoring system was used to determine the percentage of patients likely to experience PRLSRT ≥ 25% under a hypothetical 10 Fx scheme at each scoring interval. The hypothetical median PRLSRT for each scoring interval was also reported.

This project qualifies as exempt from IRB oversight due to the nature of the dataset, as the use of de-identified data for research purposes complies with ethical standards and does not require specific ethics approval.

### Statistical analysis

Statistical analyses were performed using SPSS version 27. Univariate evaluations were conducted using ANOVA, *t*-tests, or Chi-square tests. Kaplan–Meier curves with log-rank tests and Cox regression models were used to analyze survival outcomes, identify hazard ratios, and determine factors influencing patient survival. Binary logistic regression was applied to assess the likelihood of RT completion and PRLSRT > 25% in a theoretical 30 Gy/10 Fx dose regimen, with model evaluation performed using the Hosmer–Lemeshow test. Overall survival and mortality rates were calculated, and the hazard ratios (HR) for Cox regression and odds ratios (OR) for binary logistic regression were reported with 95% confidence interval (CI). Statistical significance was set at an alpha of 0.05, with *p*-values < 0.05 considered significant.

## Results

Between 2010 and 2016, a total of 50,929 patients underwent palliative RT for osseous metastases. The cohort’s median age was 66 years, with 38.8% of patients over 70. Caucasian patients comprised 84.1% of the cohort, and nonsmall cell lung cancer (NSCLC) was the most common primary cancer, accounting for 45.2% of cases. In 56.6% of patients, the bones were the only metastatic site. The majority (65.4%) received systemic chemotherapy, while 8.4% underwent surgical intervention. Regarding RT technique, 77.9% of patients received EBRT (NOS), 17.6% received 3D conformal RT, 3.3% received IMRT, 0.8% received 2D RT, and 0.3% received an unknown or other RT technique.

RT dose fractionation was stratified based on patient demographics and various characteristics, as outlined in **Table 1**. **Supplementary Table S1** presents demographic stratification by primary tumor site. The most common RT regimen was 3 Gy/Fx (60.1%), while the least common was 8 Gy/Fx (7.0%).

**Table 1:** Patient demographics by dose fractionation.

Variable (*n*, range)	Total cohort (50,929)		Dosage per fraction (variable; *n*, Col%)							
			2.5 Gy/Fx (9,518, 18.7%)		3 Gy/Fx (30,624, 60.1%)		4 Gy/Fx (7,201, 14.1%)		8 Gy/Fx (3,586, 7.0%)	
Age	66	8–90	66	18–90	66	8–90	67	14–90	67	11–90
> 70	19,765	(38.8%)	3,526	(37.0%)	11,695	(38.2%)	2,980	(41.4%)	1,564	(43.6%)
Race										
White	42,821	(84.1%)	8,163	(85.8%)	25,636	(83.7%)	6,046	(84.0%)	2,976	(83.0%)
Black	5,993	(11.8%)	1,014	(10.7%)	3,752	(12.3%)	797	(11.1%)	430	(12.0%)
Other	2,115	(4.2%)	341	(3.6%)	1,236	(4.0%)	358	(5.0%)	180	(5.0%)
Insurance										
Private	16,161	(31.7%)	3,156	(33.2%)	9,877	(32.3%)	2,142	(29.7%)	986	(27.5%)
None	2,270	(4.5%)	389	(4.1%)	1,417	(4.6%)	311	(4.3%)	153	(4.3%)
Government	31,777	(62.4%)	5,846	(61.4%)	18,883	(61.7%)	4,641	(64.4%)	2,407	(67.1%)
Unknown	721	(1.4%)	127	(1.3%)	447	(1.5%)	107	(1.5%)	40	(1.1%)
Income										
First quartile	6,311	(13.4%)	1,120	(12.7%)	3,912	(13.9%)	830	(12.5%)	449	(13.5%)
Second quartile	8,670	(18.5%)	1,721	(19.6%)	5,156	(18.3%)	1,117	(16.8%)	676	(20.4%)
Third quartile	13,569	(28.9%)	2,544	(28.9%)	8,071	(28.6%)	1,983	(29.8%)	971	(29.3%)
Fourth quartile	18,428	(39.2%)	3,405	(38.7%)	11,074	(39.3%)	2,729	(41.0%)	1,220	(36.8%)
Charlson–Deyo Score										
0	35,544	(69.8%)	6,708	(70.5%)	21,474	(70.1%)	4,966	(69.0%)	2,396	(66.8%)
1	10,273	(20.2%)	1,965	(20.6%)	6,102	(19.9%)	1,443	(20.0%)	763	(21.3%)
2	3,434	(6.7%)	588	(6.2%)	2,058	(6.7%)	521	(7.2%)	267	(7.4%)
3 or more	1,678	(3.3%)	257	(2.7%)	990	(3.2%)	271	(3.8%)	160	(4.5%)
Primary tumor										
Breast	7,694	(15.1%)	1,721	(18.1%)	4,800	(15.7%)	798	(11.1%)	375	(10.5%)
Head and neck	342	(0.7%)	39	(0.4%)	208	(0.7%)	58	(0.8%)	37	(1.0%)
Upper GI	4,332	(8.5%)	679	(7.1%)	2,541	(8.3%)	718	(10.0%)	394	(11.0%)
Lower GI	1,147	(2.3%)	187	(2.0%)	674	(2.2%)	190	(2.6%)	96	(2.7%)
GI NOS	254	(0.5%)	40	(0.4%)	147	(0.5%)	51	(0.7%)	16	(0.4%)
Lung (SC)	2,484	(4.9%)	421	(4.4%)	1,436	(4.7%)	408	(5.7%)	219	(6.1%)
Lung (NSC)	23,034	(45.2%)	4,144	(43.5%)	13,686	(44.7%)	3,486	(48.4%)	1,718	(47.9%)
Skin	378	(0.7%)	62	(0.7%)	222	(0.7%)	71	(1.0%)	23	(0.6%)
Gynecologic	558	(1.1%)	100	(1.1%)	325	(1.1%)	85	(1.2%)	48	(1.3%)
Genitourinary	4,859	(9.5%)	894	(9.4%)	2,922	(9.5%)	724	(10.1%)	319	(8.9%)
Prostate	5,501	(10.8%)	1,160	(12.2%)	3,446	(11.3%)	573	(8.0%)	322	(9.0%)
Endocrine	346	(0.7%)	71	(0.7%)	217	(0.7%)	39	(0.5%)	19	(0.5%)
T stage										
T4	8,865	(20.4%)	1,552	(19.1%)	5,347	(20.4%)	1,289	(21.0%)	677	(22.2%)
N stage										
N3	2,459	(5.0%)	393	(4.3%)	1,447	(4.9%)	390	(5.6%)	229	(6.6%)
Met involvement										
Bone only	28,825	(56.6%)	5,828	(61.2%)	17,601	(57.5%)	3,553	(49.3%)	1,843	(51.4%)
Bone w/brain	2,104	(4.1%)	407	(4.3%)	1,259	(4.1%)	307	(4.3%)	131	(3.7%)
Bone w/liver	6,128	(12.0%)	1,105	(11.6%)	3,522	(11.5%)	1,006	(14.0%)	495	(13.8%)
Bone w/lung	5,937	(11.7%)	1,112	(11.7%)	3,507	(11.5%)	900	(12.5%)	418	(11.7%)
Bone w/other	1,141	(2.2%)	94	(1.0%)	750	(2.4%)	201	(2.8%)	96	(2.7%)
Bone w/multiple	6,794	(13.3%)	972	(10.2%)	3,985	(13.0%)	1,234	(17.1%)	603	(16.8%)
Chemotherapy										
Yes	33,323	(65.4%)	6,537	(68.7%)	20,262	(66.2%)	4,441	(61.7%)	2,083	(58.1%)
Surgery										
Yes	4,253	(8.4%)	927	(9.7%)	2,533	(8.3%)	522	(7.2%)	271	(7.6%)

The median survival for all patients was 6.4 months from the first treatment intervention and 5.7 months after initiating palliative RT. Patients with breast, endocrine, and prostate cancer had the longest median survival times of 25.0, 21.5, and 21.4 months, respectively, from the start of the first intervention. In contrast, those with gastrointestinal not-otherwise-specified (GI NOS), upper GI, and NSCLC had the shortest median survival times, at 2.5, 3.5, and 3.9 months, respectively.

Patients with only metastatic bone lesions had a significantly better prognosis than those with metastases in additional organ systems, with median survival times of 8.8 and 3.5 months, respectively (**Supplementary Table S2**). Approximately one in five patients died within 30 days of starting RT. Among fractionation schemes, patients receiving 2.5 Gy/Fx had the highest mortality rate during RT (1.4%), while those receiving 8 Gy/Fx had the highest mortality rate within 90 days of completing RT (46.7%), as shown in **Supplementary Table S3**.

**Figure 1:**
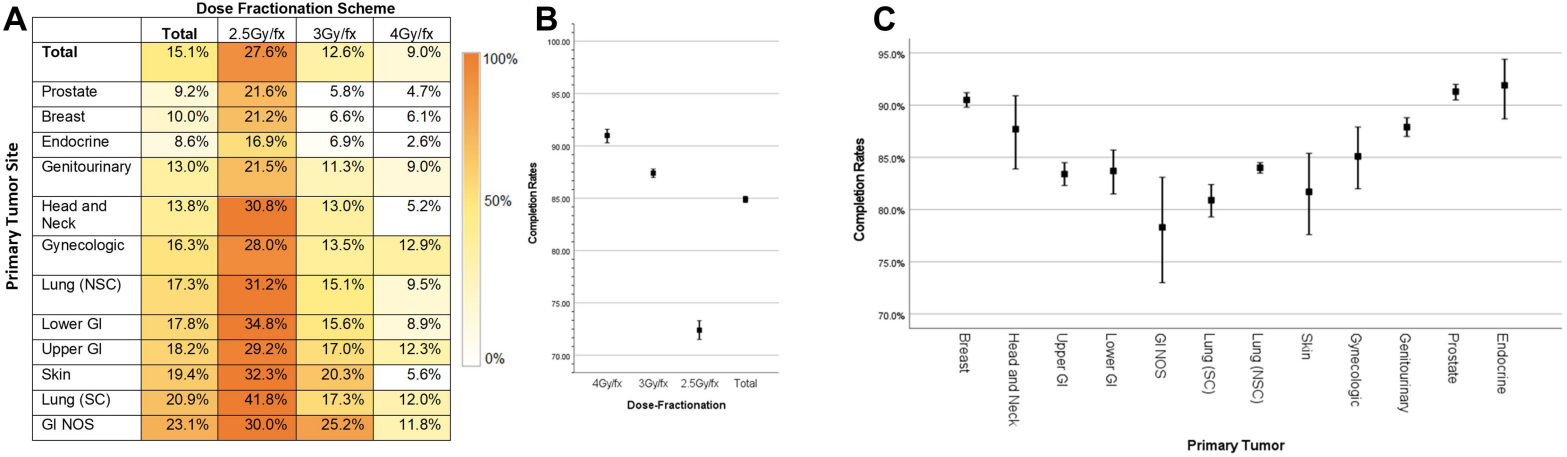
Incompletion rates by dose fractionation and primary site **(A–C)**.

Excluding the 3,586 patients who received 8 Gy/Fx, 15.1% of patients experienced incomplete radiation courses. Among them, 27.6% of patients receiving 2.5 Gy/Fx, 12.6% of patients receiving 3 Gy/Fx, and 9.0% of patients receiving 4 Gy/Fx did not complete their prescribed treatment (**Figure 1**). Patients with primary GI NOS tumors or small-cell lung cancer (SCLC) receiving 2.5Gy/Fx had the highest incompletion rates (30.0% and 41.8%, respectively); whereas those with prostate or endocrine primary tumors receiving 4 Gy/Fx had the lowest rates (4.7% and 2.6%, respectively).

Binary logistic regression analysis identified age ≥ 70, race, lower income quartile, and lower Gy/Fx as significant factors associated with incomplete RT courses. Patients aged ≥ 70 were more likely to experience incomplete RT (OR, 1.36; *p* < 0.001). Additionally, those receiving 2.5 Gy/Fx had a higher likelihood of incomplete RT compared to those receiving 4 Gy/Fx (HR, 3.780; *p* < 0.001) (**Supplementary Table S4**).

**Figure 2:**
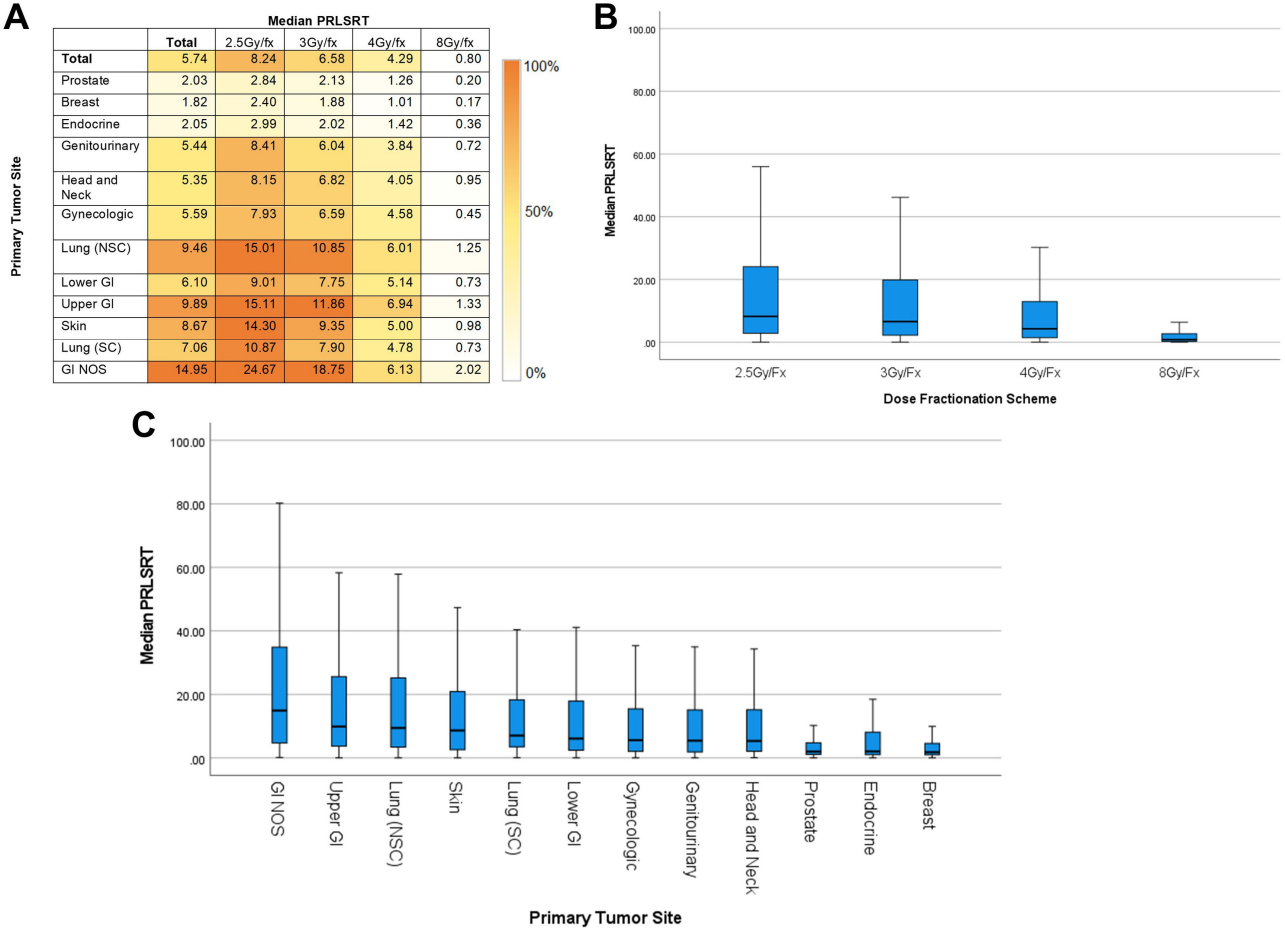
Median PRLSRT by dose fractionation and primary tumor site **(A–C)**.

Median PRLSRT was positively correlated with the total number of anticipated fractions per the standard scheme, as shown in **Figure 2**. Patients undergoing 2.5 Gy/Fx had a median PRLSRT of 8.24%, whereas those receiving 8 Gy/Fx had a median PRLSRT of 0.80%. Among patients treated with 8 Gy/Fx, those with breast cancer had the lowest median PRLSRT (0.17%), while patients with GI NOS receiving 2.5 Gy/Fx had the highest median PRLSRT (24.67%).

Patients with more comorbidities, older age, metastatic involvement in multiple organ systems, and lower dose per fraction were more likely to have proportionally higher PRLSRTs. These were stratified into four groups: PRLSRT < 10%, 10% to < 25%, 25% to < 50%, and ≥ 50% (**Supplementary Table S5**). Additionally, a scatter plot was generated to illustrate the correlation between PRLSRT and incompletion rates.

**Figure 3:**
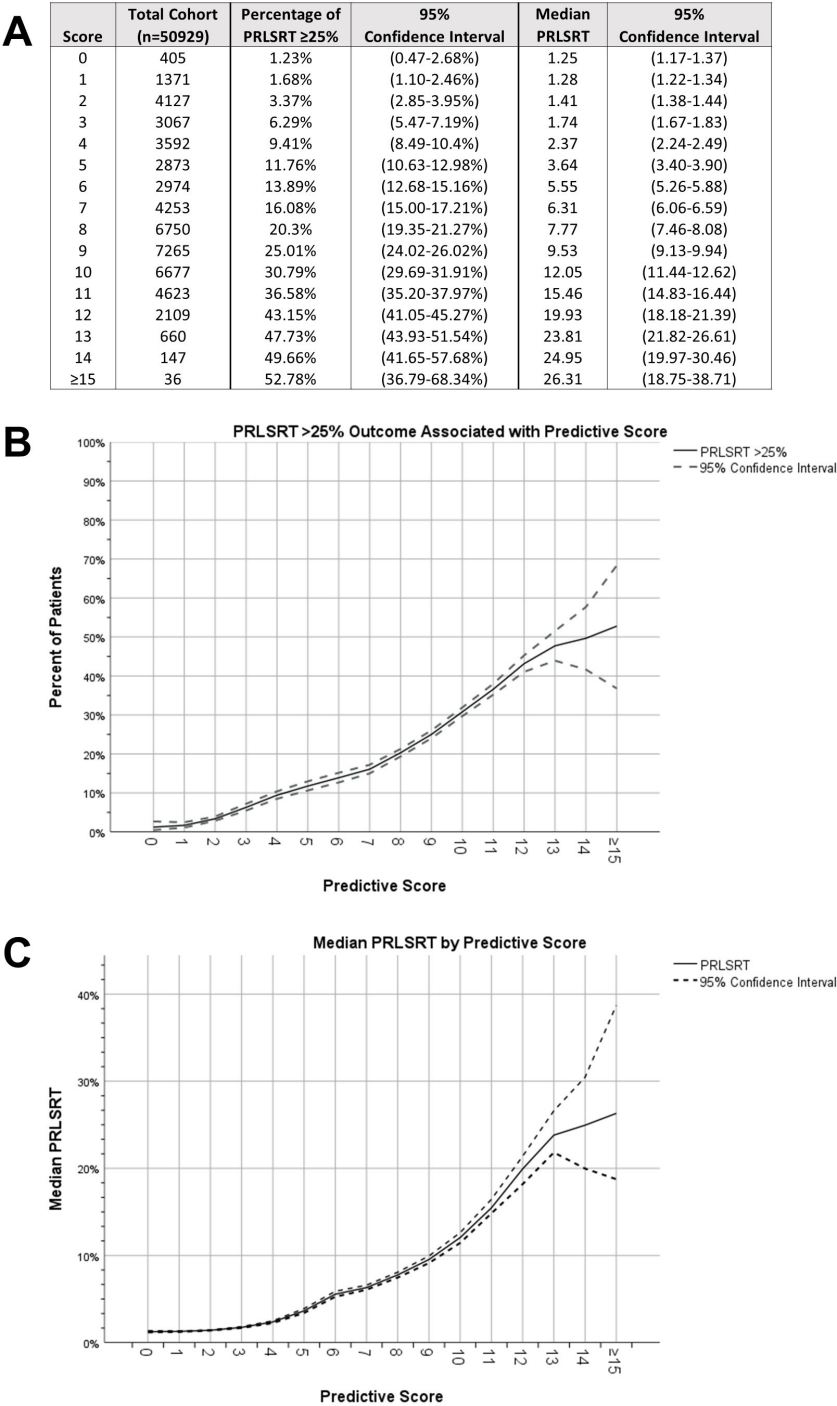
Percentage of patients with PRLSRT ≥ 25% by predicted score **(A–C)**.

Binary logistic regression identified factors that may affect the likelihood of a patient experiencing ≥ 25% PRLSRT in a hypothetical 10 Fx course. Significant factors included age, primary tumor site, bone metastasis location, metastatic organ involvement, and Charlson–Deyo Score (**Supplementary Table S6**). Using these variables, a predicted score was developed and validated in an unselected cohort (**Table 2**). The tested and validated cohorts demonstrated a fair predictive model (with ROC curve analysis yielding AUC values of 0.702 and 0.713, respectively). With a score of 9, 25.01% of patients were predicted to have a PRLSRT ≥ 25%, with a hypothesized median PRLSRT of 9.53% when undergoing a 30-Gy/10-Fx regimen (**Figure 3**).

**Table 2:** Score Assignment based on patient demographics.

	Predictive scoring for PRLSRT ≥ 25% outcomes					
**Scoring points assigned**	**9**	GI NOS				
	**8**					
	**7**	Upper GI				
	**6**	NSCLC				
	**5**	Skin GYN				
	**4**	SCLC				
	**3**	HNC Lower GI GU				
	**2**		Spine Other	Age ≥ 70	Total ≥ 3	Bone +2 system
	**1**	Endocrine	Shoulder Pelvis		Total = 1 Total = 2	Bone +1 system
	**0**	Breast Prostate	Ribs Extremity	Age < 70	Total = 0	Bone only
		***Primary tumor***	***Met location***	***Age***	***CDS score***	***Met organ involvement***
		**Patient demographics**				

## Discussion

Bone metastases are an ominous sign in many primary tumor sites and are associated with poor patient prognosis (11–13). RT can improve the quality of life for patients with limited life expectancy (2, 14). However, RT regimens should be carefully selected to minimize treatment burden while maximizing benefits (3, 15–17). In this study, we evaluated patient survival, RT completion rates, and PRLSRT. We also analyzed socioeconomic demographics to identify risk factors for incomplete RT and developed a predictive model to assist physicians in selecting appropriate palliative RT regimens for patients with bone metastases.

Our study found that patients with fewer comorbidities and a more limited metastatic organ burden were more likely to undergo protracted treatments compared to those receiving 8 Gy/1 Fx. Additionally, patients with primary cancers associated with improved prognoses were increasingly likely to receive protracted treatments. This trend aligns with multiple studies; despite single-fraction RT offering noninferior pain control and similar toxicity rates, physicians may hesitate due to concerns about retreatment in patients with longer predicted survival (15, 18, 19). However, our findings highlight an underutilization of single-fraction treatments in the USA, despite a median overall survival of less than 6 months from RT initiation and 15.1% of patients receiving an incomplete multifraction regimen (20).

In addition, our study suggests that physicians tend to overestimate patient survival rates, particularly for those with only weeks to live. This inaccuracy decreases as survival extends beyond 6 months. Our reported 30-day mortality rate—approximately one in five patients—aligns with published data, and the median survival for the total cohort was under 6 months from RT initiation (7, 8). Given that palliative RT may take weeks for its therapeutic benefits to manifest, it is crucial for physicians to ensure that patients experience relief from their intervention, especially considering their high mortality rates (16, 17). From this perspective, we advocate for the use of single-fraction palliative RT to minimize treatment burden for those with the poorest predicted prognoses.

Patients who do not complete their RT regimens may experience worse outcomes, including reduced local control, increased healthcare costs due to retreatment, the potential development of radioresistant tumors, and higher mortality (21). Incomplete radiation may also indicate that the prescribed treatment was too long, the patient was too ill, had limited financial or social resources, or that the burden of treatment outweighed its perceived benefit. Patients with primary site cancers and poor prognoses are less likely to complete their treatment, and low-dose per fraction schemas may increase the risk of partial treatment. Incomplete RT courses were more common in patients aged 70 or older, had lower incomes, were white, or were undergoing protracted regimens. Although older age has been associated with increased RT incompletion rates, further studies are needed to assess additional risk factors. While our study data do not allows for a comprehensive evaluation of the underlying causes, patients with poorer functional status and advanced age may be more vulnerable to treatment incompletion. Additionally, since RT completion is influenced by patients’ socioeconomic stability, the burden of protracted treatments is greater for those with lower incomes. Finally, physicians may choose to truncate an RT regimen as a patient’s clinical course evolves over time.

In this study, PRLSRT was used to assess the efficacy of palliative treatments concerning patient mortality. Gripp et al. proposed PRLSRT as a metric for evaluating palliative RT utilization in end-stage cancer patients. Although their patient cohort differs significantly from ours, we found this tool valuable in encapsulating the therapeutic benefit and treatment burden of palliative interventions retrospectively, even without patient-reported quality of life metrics (22). By utilizing PRLSRT, large-scale patient population analyses can be conducted to support further prospective studies and assess practice trends over time.

Previously published studies on PRLSRT outcomes suggest improvements in practice trends. Abdelhakiem et al. specifically evaluated breast cancer patients with bone metastases from 2004 to 2013 using NCDB data and found a median PRLSRT of 2.5% (23). Our study indicates further improvement in practice patterns within a similar cohort, as the median PRLSRT for breast cancer patients with bone metastases from 2010 to 2016 was 1.82%. This enhancement in palliative metrics aligns with ASTRO’s 2013 Choosing Wisely recommendations, which advocate for shorter palliative RT courses and emphasize single-fraction treatments for patients with transportation difficulties or poor functional status over protracted regimens. When stratifying the total cohort by PRLSRT, we found that patients with the highest PRLSRTs were older, had more comorbidities, and had metastases in multiple organ systems. Additionally, the location of the treated bone lesion was statistically significant, with patients receiving palliative RT to the extremities having better outcomes than those with spine lesions.

Although speculative, the observed difference in outcomes may be multifactorial. Patients with spinal involvement may present with symptoms of compression rather than pain, may be more likely to experience worsened side effects with higher doses per fractionation, and tend to have a poorer prognosis compared to those with extremity lesions. Additionally, clinicians may face limitations in using single-fraction treatments due to a greater disease burden at diagnosis or anatomical constraints. Intuitively, we found that patients with higher rates of PRLSRT were more likely to receive an incomplete treatment course of RT, as significant deterioration or death during treatment would inevitably lead to a truncated regimen.

To aid in the decision-making process for palliative radiotherapy, we developed a predictive scoring system to identify patients at higher risk of experiencing ≥ 25% PRLSRTs with the most common RT regimen, 30 Gy/10 Fx. For the 2-week treatment course, this corresponds to surviving 6 weeks after completing treatment. Our model found that the primary tumor site was the most significant predictor of poor PRLSRT outcomes; however, other factors such as patient comorbidities, age, metastatic burden, and bone met location should also be considered. The use of objective measures can help minimize physician bias, which may lead to poor palliative RT utilization. Based on our analysis, we recommend that patients with a score of ≥ 9 undergo a shorter fractionation scheme, as one in four patients with this score experienced a PRLSRT ≥ 25%. As Jones et al. state, “If radiotherapy at the end of life is to be incorporated as a quality indicator, we would recommend using the PRLSRT as the indicator with a cutoff of 10%”. This recommendation aligns well as a proposed cutoff, as the median PRLSRT for a score of 9 in this theoretical cohort was 9.53% (17). The choice of palliative RT fractionation is complex, but our scoring system may provide valuable guidance for clinicians.

While our retrospective data support the use of 8 Gy/1 Fx treatments to minimize PRLSRT and increase completion rates, we must also consider the potential risks of retreatment and late toxicities. Our dataset lacked information on pain response, logistical burden, and the financial impact of treatment on patients, making it difficult to evaluate their quality of life during and after RT. Moreover, our PRLSRT calculation was based on an estimated treatment schedule, and life expectancy was calculated from the start of RT to the last patient contact or death.

Using this retrospective data, physicians can tailor their palliative RT course selection to better suit each patient’s needs, considering overall survival, treatment logistics, and symptom control. Further research is necessary to assess the impact of dose-fractionation schedules for palliative RT on symptom control, patient socioeconomic burden, practitioner machine utilization, and perceived quality of life improvement for patients/families. Prospective studies can evaluate our predictive scoring system and address the limitations of this retrospective study by providing a more in-depth analysis of patient outcomes.

In conclusion, palliative RT requires a delicate balance, considering various factors that influence patient outcomes and quality of life. Our retrospective data provide valuable insight into the benefits of 8 Gy/1 Fx treatments, but further research is necessary to fully determine the best approach for palliative RT. Additional studies can help identify the optimal dose-fractionation schedule and assess its impact on patient’s lives, guiding physicians in selecting the most appropriate treatment course.

## Data Availability

The original contributions presented in the study are included in the article/**Supplementary Materials**, further inquiries can be directed to the corresponding author/s.
