# Supplementary material for: Implications of primary tumor site and fraction size on outcomes of palliative radiation for osseous metastases
Source: Front Oncol. 2025 Mar 31;15:1432916. doi: 10.3389/fonc.2025.1432916 (PMC11994703; doi:10.3389/fonc.2025.1432916)

**Supplemental Table 1: Patient Demographics by Primary Tumor Site**

| **Variable (n, range)**  **Variable (n, Col%)** | | **Total Cohort**  (50929) | | **Primary Tumor Site** | | | | | | | | | | | | | | | | | | | | | | | |
| --- | --- | --- | --- | --- | --- | --- | --- | --- | --- | --- | --- | --- | --- | --- | --- | --- | --- | --- | --- | --- | --- | --- | --- | --- | --- | --- | --- |
|  |  |  |  | **Breast**  (7694, 15.1%) | | **Head and Neck**  (342, 0.7%) | | **Upper GI**  (4332, 8.5%) | | **Lower GI**  (1147, 2.3%) | | **GI NOS**  (254, 0.5%) | | **Lung (SC)**  (2484, 4.9%) | | **Lung (NSC)**  (23034, 45.2%) | | **Skin**  (378, 0.7%) | | **Gynecologic**  (558, 1.1%) | | **Genitourinary**  (4859, 9.5%) | | **Prostate**  (5501, 10.8%) | | **Endocrine**  (346, 0.7%) | |
| **Age** | | 66 | 8 - 90 | 61 | 21 - 90 | 62 | 18 - 90 | 63 | 15 - 90 | 62 | 20 - 90 | 67 | 19 - 90 | 66 | 26 - 90 | 68 | 18 - 90 | 64 | 21 - 90 | 61 | 21 - 90 | 66 | 11 - 90 | 69 | 33 - 90 | 65 | 8 - 90 |
|  | >70 | 19765 | (38.8%) | 2090 | (27.2%) | 90 | (26.3%) | 1273 | (29.4%) | 335 | (29.2%) | 105 | (41.3%) | 922 | (37.1%) | 10031 | (43.5%) | 130 | (34.4%) | 140 | (25.1%) | 1856 | (38.2%) | 2665 | (48.4%) | 128 | (37.0%) |
| **Race** | |  |  |  |  |  |  |  |  |  |  |  |  |  |  |  |  |  |  |  |  |  |  |  |  |  |  |
|  | White | 42821 | (84.1%) | 6243 | (81.1%) | 273 | (79.8%) | 3559 | (82.2%) | 914 | (79.7%) | 210 | (82.7%) | 2258 | (90.9%) | 19761 | (85.8%) | 360 | (95.2%) | 433 | (77.6%) | 4277 | (88.0%) | 4266 | (77.5%) | 267 | (77.2%) |
|  | Black | 5993 | (11.8%) | 1142 | (14.8%) | 50 | (14.6%) | 512 | (11.8%) | 167 | (14.6%) | 29 | (11.4%) | 173 | (7.0%) | 2348 | (10.2%) | 8 | (2.1%) | 93 | (16.7%) | 415 | (8.5%) | 993 | (18.1%) | 63 | (18.2%) |
|  | Other | 2115 | (4.2%) | 309 | (4.0%) | 19 | (5.6%) | 261 | (6.0%) | 66 | (5.8%) | 15 | (5.9%) | 53 | (2.1%) | 925 | (4.0%) | 10 | (2.6%) | 32 | (5.7%) | 167 | (3.4%) | 242 | (4.4%) | 16 | (4.6%) |
| **Insurance** | |  |  |  |  |  |  |  |  |  |  |  |  |  |  |  |  |  |  |  |  |  |  |  |  |  |  |
|  | Private | 16161 | (31.7%) | 3047 | (39.6%) | 108 | (31.6%) | 1578 | (36.4%) | 467 | (40.7%) | 79 | (31.1%) | 694 | (27.9%) | 6653 | (28.9%) | 137 | (36.2%) | 222 | (39.8%) | 1670 | (34.4%) | 1375 | (25.0%) | 131 | (37.9%) |
|  | None | 2270 | (4.5%) | 428 | (5.6%) | 26 | (7.6%) | 229 | (5.3%) | 82 | (7.1%) | 13 | (5.1%) | 110 | (4.4%) | 808 | (3.5%) | 19 | (5.0%) | 30 | (5.4%) | 191 | (3.9%) | 320 | (5.8%) | 14 | (4.0%) |
|  | Government | 31777 | (62.4%) | 4106 | (53.4%) | 205 | (59.9%) | 2470 | (57.0%) | 581 | (50.7%) | 161 | (63.4%) | 1649 | (66.4%) | 15232 | (66.1%) | 218 | (57.7%) | 296 | (53.0%) | 2929 | (60.3%) | 3736 | (67.9%) | 194 | (56.1%) |
|  | Unknown | 721 | (1.4%) | 113 | (1.5%) | 3 | (0.9%) | 55 | (1.3%) | 17 | (1.5%) | 1 | (0.4%) | 31 | (1.2%) | 341 | (1.5%) | 4 | (1.1%) | 10 | (1.8%) | 69 | (1.4%) | 70 | (1.3%) | 7 | (2.0%) |
| **Income** | |  |  |  |  |  |  |  |  |  |  |  |  |  |  |  |  |  |  |  |  |  |  |  |  |  |  |
|  | 1st Quartile | 6311 | (13.4%) | 944 | (13.6%) | 45 | (14.3%) | 594 | (14.7%) | 159 | (14.8%) | 31 | (13.1%) | 343 | (14.8%) | 2725 | (12.7%) | 36 | (10.3%) | 74 | (14.1%) | 571 | (12.8%) | 754 | (15.2%) | 35 | (11.1%) |
|  | 2nd Quartile | 8670 | (18.5%) | 1252 | (18.0%) | 57 | (18.1%) | 704 | (17.4%) | 202 | (18.8%) | 36 | (15.2%) | 507 | (21.8%) | 3941 | (18.4%) | 64 | (18.3%) | 95 | (18.1%) | 848 | (19.1%) | 902 | (18.2%) | 62 | (19.7%) |
|  | 3rd Quartile | 13569 | (28.9%) | 1912 | (27.5%) | 101 | (32.1%) | 1160 | (28.7%) | 326 | (30.4%) | 67 | (28.3%) | 691 | (29.7%) | 6269 | (29.2%) | 95 | (27.2%) | 149 | (28.4%) | 1311 | (29.5%) | 1400 | (28.2%) | 88 | (27.9%) |
|  | 4th Quartile | 18428 | (39.2%) | 2837 | (40.8%) | 112 | (35.6%) | 1587 | (39.2%) | 387 | (36.0%) | 103 | (43.5%) | 784 | (33.7%) | 8507 | (39.7%) | 154 | (44.1%) | 206 | (39.3%) | 1720 | (38.7%) | 1901 | (38.3%) | 130 | (41.3%) |
| **Charlson Deyo Score** | |  |  |  |  |  |  |  |  |  |  |  |  |  |  |  |  |  |  |  |  |  |  |  |  |  |  |
|  | 0 | 35544 | (69.8%) | 6242 | (81.1%) | 265 | (77.5%) | 2836 | (65.5%) | 890 | (77.6%) | 165 | (65.0%) | 1448 | (58.3%) | 15107 | (65.6%) | 298 | (78.8%) | 437 | (78.3%) | 3485 | (71.7%) | 4106 | (74.6%) | 265 | (76.6%) |
|  | 1 | 10273 | (20.2%) | 1028 | (13.4%) | 49 | (14.3%) | 994 | (22.9%) | 176 | (15.3%) | 62 | (24.4%) | 684 | (27.5%) | 5276 | (22.9%) | 50 | (13.2%) | 87 | (15.6%) | 933 | (19.2%) | 876 | (15.9%) | 58 | (16.8%) |
|  | 2 | 3434 | (6.7%) | 295 | (3.8%) | 19 | (5.6%) | 286 | (6.6%) | 56 | (4.9%) | 18 | (7.1%) | 233 | (9.4%) | 1835 | (8.0%) | 20 | (5.3%) | 24 | (4.3%) | 288 | (5.9%) | 346 | (6.3%) | 14 | (4.0%) |
|  | 3 or more | 1678 | (3.3%) | 129 | (1.7%) | 9 | (2.6%) | 216 | (5.0%) | 25 | (2.2%) | 9 | (3.5%) | 119 | (4.8%) | 816 | (3.5%) | 10 | (2.6%) | 10 | (1.8%) | 153 | (3.1%) | 173 | (3.1%) | 9 | (2.6%) |
| **T Stage** | |  |  |  |  |  |  |  |  |  |  |  |  |  |  |  |  |  |  |  |  |  |  |  |  |  |  |
|  | T4 | 8865 | (20.4%) | 1417 | (20.4%) | 92 | (28.0%) | 356 | (9.2%) | 172 | (15.6%) | 0 | (0.0%) | 667 | (30.6%) | 5142 | (25.9%) | 34 | (10.1%) | 31 | (6.8%) | 401 | (10.4%) | 495 | (12.2%) | 58 | (17.3%) |
| **N Stage** | |  |  |  |  |  |  |  |  |  |  |  |  |  |  |  |  |  |  |  |  |  |  |  |  |  |  |
|  | N3 | 2459 | (5.0%) | 211 | (2.9%) | 14 | (4.2%) | 62 | (1.5%) | 1 | (0.1%) | 0 | (0.0%) | 233 | (9.7%) | 1901 | (8.5%) | 9 | (2.6%) | 2 | (0.4%) | 26 | (0.5%) | 0 | (0.0%) | 0 | (0.0%) |
| **Metastatic Involvement** | |  |  |  |  |  |  |  |  |  |  |  |  |  |  |  |  |  |  |  |  |  |  |  |  |  |  |
|  | Bone Only | 28825 | (56.6%) | 4795 | (62.3%) | 186 | (54.4%) | 2064 | (47.6%) | 278 | (24.2%) | 86 | (33.9%) | 882 | (35.5%) | 12319 | (53.5%) | 131 | (34.7%) | 245 | (43.9%) | 2736 | (56.3%) | 4903 | (89.1%) | 200 | (57.8%) |
|  | Bone w/Brain | 2104 | (4.1%) | 202 | (2.6%) | 7 | (2.0%) | 62 | (1.4%) | 7 | (0.6%) | 7 | (2.8%) | 138 | (5.6%) | 1551 | (6.7%) | 16 | (4.2%) | 11 | (2.0%) | 60 | (1.2%) | 37 | (0.7%) | 6 | (1.7%) |
|  | Bone w/Liver | 6128 | (12.0%) | 765 | (9.9%) | 33 | (9.6%) | 929 | (21.4%) | 345 | (30.1%) | 59 | (23.2%) | 831 | (33.5%) | 2708 | (11.8%) | 43 | (11.4%) | 41 | (7.3%) | 287 | (5.9%) | 70 | (1.3%) | 17 | (4.9%) |
|  | Bone w/Lung | 5937 | (11.7%) | 927 | (12.0%) | 54 | (15.8%) | 452 | (10.4%) | 87 | (7.6%) | 36 | (14.2%) | 144 | (5.8%) | 2734 | (11.9%) | 50 | (13.2%) | 151 | (27.1%) | 1051 | (21.6%) | 179 | (3.3%) | 72 | (20.8%) |
|  | Bone w/Other | 1141 | (2.2%) | 111 | (1.4%) | 5 | (1.5%) | 73 | (1.7%) | 22 | (1.9%) | 0 | (0.0%) | 32 | (1.3%) | 580 | (2.5%) | 7 | (1.9%) | 18 | (3.2%) | 90 | (1.9%) | 202 | (3.7%) | 1 | (0.3%) |
|  | Bone w/Multiple | 6794 | (13.3%) | 894 | (11.6%) | 57 | (16.7%) | 752 | (17.4%) | 408 | (35.6%) | 66 | (26.0%) | 457 | (18.4%) | 3142 | (13.6%) | 131 | (34.7%) | 92 | (16.5%) | 635 | (13.1%) | 110 | (2.0%) | 50 | (14.5%) |
| **Dosage per Fraction** | |  |  |  |  |  |  |  |  |  |  |  |  |  |  |  |  |  |  |  |  |  |  |  |  |  |  |
|  | 2.5Gy/Fx | 9518 | (18.7%) | 1721 | (22.4%) | 39 | (11.4%) | 679 | (15.7%) | 187 | (16.3%) | 40 | (15.7%) | 421 | (16.9%) | 4144 | (18.0%) | 62 | (16.4%) | 100 | (17.9%) | 894 | (18.4%) | 1160 | (21.1%) | 71 | (20.5%) |
|  | 3Gy/Fx | 30624 | (60.1%) | 4800 | (62.4%) | 208 | (60.8%) | 2541 | (58.7%) | 674 | (58.8%) | 147 | (57.9%) | 1436 | (57.8%) | 13686 | (59.4%) | 222 | (58.7%) | 325 | (58.2%) | 2922 | (60.1%) | 3446 | (62.6%) | 217 | (62.7%) |
|  | 4Gy/Fx | 7201 | (14.1%) | 798 | (10.4%) | 58 | (17.0%) | 718 | (16.6%) | 190 | (16.6%) | 51 | (20.1%) | 408 | (16.4%) | 3486 | (15.1%) | 71 | (18.8%) | 85 | (15.2%) | 724 | (14.9%) | 573 | (10.4%) | 39 | (11.3%) |
|  | 8Gy/Fx | 3586 | (7.0%) | 375 | (4.9%) | 37 | (10.8%) | 394 | (9.1%) | 96 | (8.4%) | 16 | (6.3%) | 219 | (8.8%) | 1718 | (7.5%) | 23 | (6.1%) | 48 | (8.6%) | 319 | (6.6%) | 322 | (5.9%) | 19 | (5.5%) |
| **Chemotherapy** | |  |  |  |  |  |  |  |  |  |  |  |  |  |  |  |  |  |  |  |  |  |  |  |  |  |  |
|  | Yes | 33323 | (65.4%) | 6451 | (83.8%) | 220 | (64.3%) | 2487 | (57.4%) | 772 | (67.3%) | 115 | (45.3%) | 1909 | (76.9%) | 13049 | (56.7%) | 217 | (57.4%) | 346 | (62.0%) | 2827 | (58.2%) | 4739 | (86.1%) | 191 | (55.2%) |
| **Surgery** | |  |  |  |  |  |  |  |  |  |  |  |  |  |  |  |  |  |  |  |  |  |  |  |  |  |  |
|  | Yes | 4253 | (8.4%) | 1174 | (15.3%) | 47 | (13.7%) | 86 | (2.0%) | 222 | (19.4%) | 0 | (0.0%) | 13 | (0.5%) | 262 | (1.1%) | 60 | (15.9%) | 161 | (28.9%) | 1781 | (36.7%) | 250 | (4.5%) | 197 | (56.9%) |

**Supplemental Table 2: Cox Regression with Characteristics affecting Overall Survival**

|  | | Median Overall  Survival (Months) | Median Survival  from RT  (Months) | **Univariate Analysis** | | | | **Multivariate Analysis** | | | |
| --- | --- | --- | --- | --- | --- | --- | --- | --- | --- | --- | --- |
|  |  |  |  | Hazard  Ratio | Lower  CI | Upper  CI | p-value | Hazard  Ratio | Lower  CI | Upper  CI | p-value |
| **Age** | | - | - | 1.017 | 1.016 | 1.017 | <0.001 | 1.015 | 1.014 | 1.016 | <0.001 |
| **Race** | | 6.44 | 5.74 |  |  |  |  |  |  |  |  |
|  | White | 6.21 | 5.51 | (ref) | (ref) | (ref) | <0.001 | (ref) | (ref) | (ref) | <0.001 |
|  | Black | 7.72 | 6.83 | 0.883 | 0.854 | 0.913 | <0.001 | 0.972 | 0.939 | 1.007 | 0.113 |
|  | Other | 9.4 | 8.46 | 0.742 | 0.701 | 0.786 | <0.001 | 0.715 | 0.675 | 0.757 | <0.001 |
| **Insurance** | |  |  |  |  |  |  |  |  |  |  |
|  | Private | 9.39 | 8.69 | (ref) | (ref) | (ref) | <0.001 | (ref) | (ref) | (ref) | <0.001 |
|  | None | 6.57 | 5.74 | 1.133 | 1.072 | 1.197 | <0.001 | 1.225 | 1.159 | 1.294 | <0.001 |
|  | Government | 5.29 | 4.65 | 1.357 | 1.326 | 1.389 | <0.001 | 1.130 | 1.100 | 1.161 | <0.001 |
|  | Unknown | 5.6 | 4.86 | 1.318 | 1.204 | 1.444 | <0.001 | 1.197 | 1.093 | 1.312 | <0.001 |
| **Income Quartile** | |  |  |  |  |  |  |  |  |  |  |
|  | 4th Quartile | 6.65 | 5.91 | (ref) | (ref) | (ref) | <0.001 | (ref) | (ref) | (ref) | <0.001 |
|  | 3rd Quartile | 5.92 | 5.28 | 1.095 | 1.069 | 1.121 | <0.001 | 1.086 | 1.058 | 1.115 | <0.001 |
|  | 2nd Quartile | 5.72 | 4.98 | 1.111 | 1.080 | 1.142 | <0.001 | 1.108 | 1.075 | 1.142 | <0.001 |
|  | 1st Quartile | 5.97 | 5.2 | 1.077 | 1.044 | 1.111 | <0.001 | 1.096 | 1.058 | 1.134 | <0.001 |
| **Charlson-Deyo Score** | |  |  |  |  |  |  |  |  |  |  |
|  | 0 | 7.71 | 6.95 | (ref) | (ref) | (ref) | <0.001 | (ref) | (ref) | (ref) | <0.001 |
|  | 1 | 4.74 | 4.09 | 1.377 | 1.341 | 1.414 | <0.001 | 1.201 | 1.170 | 1.233 | <0.001 |
|  | 2 | 3.78 | 3.3 | 1.559 | 1.496 | 1.625 | <0.001 | 1.322 | 1.268 | 1.378 | <0.001 |
|  | 3 or more | 2.95 | 2.56 | 1.739 | 1.641 | 1.843 | <0.001 | 1.442 | 1.360 | 1.529 | <0.001 |
| **Primary Site** | |  |  |  |  |  |  |  |  |  |  |
|  | Breast | 24.99 | 24.36 | (ref) | (ref) | (ref) | <0.001 | (ref) | (ref) | (ref) | <0.001 |
|  | Head and Neck | 6.04 | 4.89 | 2.789 | 2.485 | 3.130 | <0.001 | 2.652 | 2.347 | 2.996 | <0.001 |
|  | Upper GI | 3.53 | 2.96 | 3.998 | 3.833 | 4.170 | <0.001 | 3.514 | 3.352 | 3.683 | <0.001 |
|  | Lower GI | 5.61 | 4.25 | 2.923 | 2.734 | 3.126 | <0.001 | 2.505 | 2.329 | 2.695 | <0.001 |
|  | GI NOS | 2.46 | 2.29 | 4.944 | 4.335 | 5.638 | <0.001 | 4.055 | 3.531 | 4.657 | <0.001 |
|  | Lung (SC) | 5.46 | 4.27 | 3.772 | 3.589 | 3.963 | <0.001 | 2.808 | 2.655 | 2.970 | <0.001 |
|  | Lung (NSC) | 3.92 | 3.56 | 3.510 | 3.402 | 3.622 | <0.001 | 3.120 | 3.011 | 3.233 | <0.001 |
|  | Skin | 4.45 | 3.66 | 2.892 | 2.583 | 3.239 | <0.001 | 2.502 | 2.207 | 2.836 | <0.001 |
|  | Gynecologic | 6.57 | 5.14 | 2.410 | 2.194 | 2.646 | <0.001 | 2.340 | 2.102 | 2.605 | <0.001 |
|  | Genitourinary | 6.83 | 5.99 | 2.255 | 2.163 | 2.350 | <0.001 | 2.281 | 2.175 | 2.392 | <0.001 |
|  | Prostate | 21.38 | 20.22 | 1.103 | 1.057 | 1.151 | <0.001 | 1.070 | 1.018 | 1.125 | 0.008 |
|  | Endocrine | 21.52 | 20.97 | 1.020 | 0.890 | 1.168 | 0.779 | 0.987 | 0.850 | 1.145 | 0.859 |
| **T Stage** | |  |  |  |  |  |  |  |  |  |  |
|  | T1-T3 | 6.63 | 5.98 | (ref) | (ref) | (ref) | (ref) | (ref) | (ref) | (ref) | (ref) |
|  | T4 | 5.15 | 4.39 | 1.190 | 1.160 | 1.222 | <0.001 | 1.077 | 1.048 | 1.106 | <0.001 |
| **N Stage** | |  |  |  |  |  |  |  |  |  |  |
|  | N0-N2 | 6.57 | 5.9 | (ref) | (ref) | (ref) | (ref) | (ref) | (ref) | (ref) | (ref) |
|  | N3 | 4.43 | 3.73 | 1.343 | 1.284 | 1.405 | <0.001 | 1.015 | 0.965 | 1.067 | 0.572 |
| **Met Involvement** | |  |  |  |  |  |  |  |  |  |  |
|  | Bone Only | 8.83 | 8.15 | (ref) | (ref) | (ref) | <0.001 | (ref) | (ref) | (ref) | <0.001 |
|  | Bone w/Brain | 4.46 | 4.2 | 1.534 | 1.462 | 1.610 | <0.001 | 1.238 | 1.174 | 1.306 | <0.001 |
|  | Bone w/Liver | 4.13 | 3.31 | 1.714 | 1.663 | 1.767 | <0.001 | 1.433 | 1.385 | 1.482 | <0.001 |
|  | Bone w/Lung | 5.61 | 4.94 | 1.310 | 1.270 | 1.352 | <0.001 | 1.172 | 1.132 | 1.213 | <0.001 |
|  | Bone w/Other | 6 | 5.3 | 1.197 | 1.113 | 1.288 | <0.001 | 1.076 | 0.991 | 1.169 | 0.082 |
|  | Bone w/Multiple | 3.54 | 2.95 | 1.754 | 1.703 | 1.806 | <0.001 | 1.517 | 1.468 | 1.567 | <0.001 |
| **Osseous Location** | |  |  |  |  |  |  |  |  |  |  |
|  | Spine | 5.61 | 4.96 | (ref) | (ref) | (ref) | <0.001 | (ref) | (ref) | (ref) | <0.001 |
|  | Shoulder | 6.01 | 5.4 | 1.030 | 0.977 | 1.086 | 0.276 | 0.851 | 0.803 | 0.902 | <0.001 |
|  | Ribs | 6.27 | 5.71 | 1.060 | 1.006 | 1.118 | 0.030 | 0.788 | 0.744 | 0.835 | <0.001 |
|  | Pelvic bones | 8.14 | 7.29 | 0.850 | 0.828 | 0.872 | <0.001 | 0.849 | 0.825 | 0.872 | <0.001 |
|  | Extremity, NOS | 9.1 | 8.38 | 0.804 | 0.774 | 0.835 | <0.001 | 0.747 | 0.717 | 0.779 | <0.001 |
|  | Other | 6.6 | 5.71 | 0.921 | 0.885 | 0.959 | <0.001 | 0.938 | 0.898 | 0.980 | 0.004 |
| **Dose Per Faction** | |  |  |  |  |  |  |  |  |  |  |
|  | 2.5Gy/Fx | 7.88 | 7.21 | (ref) | (ref) | (ref) | <0.001 | (ref) | (ref) | (ref) | <0.001 |
|  | 3Gy/Fx | 6.8 | 6.12 | 1.072 | 1.046 | 1.099 | <0.001 | 1.025 | 0.996 | 1.054 | 0.087 |
|  | 4Gy/Fx | 4.82 | 4.1 | 1.297 | 1.255 | 1.341 | <0.001 | 1.123 | 1.082 | 1.166 | <0.001 |
|  | 8Gy/Fx | 4.14 | 3.33 | 1.433 | 1.375 | 1.494 | <0.001 | 1.290 | 1.231 | 1.352 | <0.001 |

**Supplemental Table 3: Mortality associated with Dose-Fractionation**

| VARIABLE (n, n%) | Total Cohort | | **Dose per Fractionation** | | | | | | | |
| --- | --- | --- | --- | --- | --- | --- | --- | --- | --- | --- |
|  |  |  | 2.5Gy/Fx | | 3Gy/Fx | | 4Gy/Fx | | 8Gy/Fx | |
| Died on RT | 539 | 1.1% | 130 | 1.4% | 317 | 1.0% | 72 | 1.0% | 20 | 0.6% |
| 30-Day Mortality | 10268 | 20.2% | 1818 | 19.1% | 5909 | 19.3% | 1644 | 22.8% | 897 | 25.0% |
| 90-Day Mortality | 19260 | 37.8% | 3317 | 34.8% | 11153 | 36.4% | 3115 | 43.3% | 1675 | 46.7% |


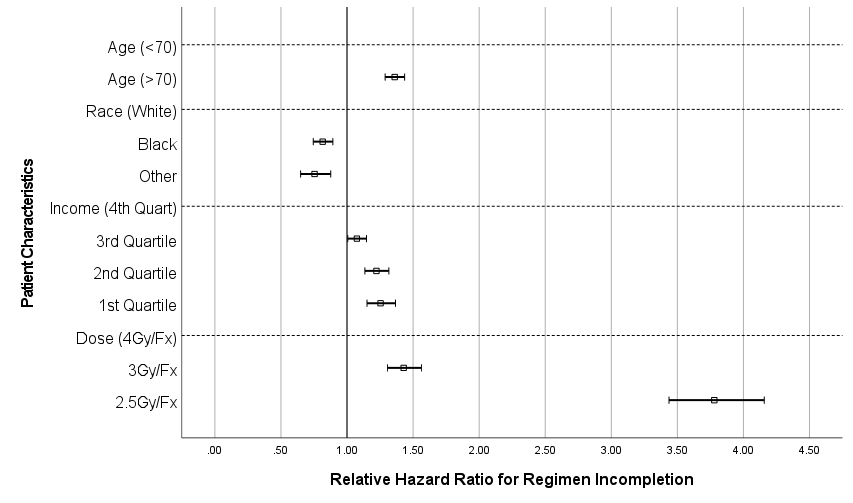
**Supplemental Table 4: Binary Logistic Regression for Factors affecting RT Regimen Incompletion (A/B)**

| **Variable** | | Incompletion  Rates | **Binary Logistic Regression** | | | |
| --- | --- | --- | --- | --- | --- | --- |
|  |  |  | Odds Ratio | Lower CI | Upper CI | p-value |
| **Age** | | 14.02% |  |  |  |  |
|  | <70 Years Old | 12.84% | (ref) | (ref) | (ref) |  |
|  | ≥70 Years Old | 15.88% | 1.361 | 1.289 | 1.436 | <0.001 |
| **Race** | |  |  |  |  |  |
|  | White | 14.51% | (ref) | (ref) | (ref) | <0.001 |
|  | Black | 11.76% | 0.816 | 0.745 | 0.893 | <0.001 |
|  | Other | 10.50% | 0.755 | 0.649 | 0.877 | <0.001 |
| **Income Quartile** | |  |  |  |  |  |
|  | 4th Quartile | 13.31% | (ref) | (ref) | (ref) | <0.001 |
|  | 3rd Quartile | 13.97% | 1.075 | 1.006 | 1.148 | 0.033 |
|  | 2nd Quartile | 15.61% | 1.223 | 1.136 | 1.317 | <0.001 |
|  | 1st Quartile | 14.97% | 1.254 | 1.152 | 1.367 | <0.001 |
| **Dose Per Faction** | |  |  |  |  |  |
|  | 4Gy/Fx | 9.04% | (ref) | (ref) | (ref) | <0.001 |
|  | 3Gy/Fx | 12.62% | 1.429 | 1.306 | 1.563 | <0.001 |
|  | 2.5Gy/Fx | 27.57% | 3.780 | 3.437 | 4.158 | <0.001 |

**Supplemental Table 5: Patient Demographics Stratified by PRLSRT**

| **Variable (n, Row%)** | | **PRLSRT Stratification** | | | | | | | |
| --- | --- | --- | --- | --- | --- | --- | --- | --- | --- |
|  |  | PRLSRT <10%  (62.5%) | | PRLSRT 10-<25%  (18.6%) | | PRLSRT 25-<50%  (11.2%) | | PRLSRT ≥50%  (7.6%) | |
| **Age** | |  |  |  |  |  |  |  |  |
|  | <70 | 20878 | (67.0%) | 5358 | (17.2%) | 2990 | (9.6%) | 1938 | (6.2%) |
|  | >70 | 10953 | (55.4%) | 4138 | (20.9%) | 2719 | (13.8%) | 1955 | (9.9%) |
| **Race** | |  |  |  |  |  |  |  |  |
|  | White | 26415 | (61.7%) | 8099 | (18.9%) | 4919 | (11.5%) | 3388 | (7.9%) |
|  | Black | 3964 | (66.1%) | 1048 | (17.5%) | 596 | (9.9%) | 385 | (6.4%) |
|  | Other | 1452 | (68.7%) | 349 | (16.5%) | 194 | (9.2%) | 120 | (5.7%) |
| **Charlson Deyo Score** | |  |  |  |  |  |  |  |  |
|  | 0 | 23538 | (66.2%) | 6176 | (17.4%) | 3488 | (9.8%) | 2342 | (6.6%) |
|  | 1 | 5742 | (55.9%) | 2181 | (21.2%) | 1404 | (13.7%) | 946 | (9.2%) |
|  | 2 | 1765 | (51.4%) | 751 | (21.9%) | 538 | (15.7%) | 380 | (11.1%) |
|  | 3 or more | 786 | (46.8%) | 388 | (23.1%) | 279 | (16.6%) | 225 | (13.4%) |
| **Primary Tumor** | |  |  |  |  |  |  |  |  |
|  | Breast | 6522 | (84.8%) | 627 | (8.1%) | 333 | (4.3%) | 212 | (2.8%) |
|  | Head and Neck | 217 | (63.5%) | 74 | (21.6%) | 36 | (10.5%) | 15 | (4.4%) |
|  | Upper GI | 2178 | (50.3%) | 1044 | (24.1%) | 667 | (15.4%) | 443 | (10.2%) |
|  | Lower GI | 699 | (60.9%) | 234 | (20.4%) | 121 | (10.5%) | 93 | (8.1%) |
|  | GI NOS | 100 | (39.4%) | 70 | (27.6%) | 43 | (16.9%) | 41 | (16.1%) |
|  | Lung (SC) | 1507 | (60.7%) | 488 | (19.6%) | 273 | (11.0%) | 216 | (8.7%) |
|  | Lung (NSC) | 11850 | (51.4%) | 5368 | (23.3%) | 3450 | (15.0%) | 2366 | (10.3%) |
|  | Skin | 208 | (55.0%) | 92 | (24.3%) | 40 | (10.6%) | 38 | (10.1%) |
|  | Gynecologic | 362 | (64.9%) | 110 | (19.7%) | 47 | (8.4%) | 39 | (7.0%) |
|  | Genitourinary | 3167 | (65.2%) | 920 | (18.9%) | 480 | (9.9%) | 292 | (6.0%) |
|  | Prostate | 4747 | (86.3%) | 421 | (7.7%) | 206 | (3.7%) | 127 | (2.3%) |
|  | Endocrine | 274 | (79.2%) | 48 | (13.9%) | 13 | (3.8%) | 11 | (3.2%) |
| **Metastatic Involvement** | |  |  |  |  |  |  |  |  |
|  | Bone Only | 19670 | (68.2%) | 4784 | (16.6%) | 2642 | (9.2%) | 1729 | (6.0%) |
|  | Bone w/Brain | 1129 | (53.7%) | 492 | (23.4%) | 266 | (12.6%) | 217 | (10.3%) |
|  | Bone w/Liver | 3305 | (53.9%) | 1308 | (21.3%) | 865 | (14.1%) | 650 | (10.6%) |
|  | Bone w/Lung | 3550 | (59.8%) | 1191 | (20.1%) | 726 | (12.2%) | 470 | (7.9%) |
|  | Bone w/Other | 709 | (62.1%) | 215 | (18.8%) | 146 | (12.8%) | 71 | (6.2%) |
|  | Bone w/Multiple | 3468 | (51.0%) | 1506 | (22.2%) | 1064 | (15.7%) | 756 | (11.1%) |
| **RT Completion** | |  |  |  |  |  |  |  |  |
|  | Incomplete | 2609 | (36.5%) | 1519 | (21.3%) | 1355 | (19.0%) | 1656 | (23.2%) |
|  | Complete | 29222 | (66.7%) | 7977 | (18.2%) | 4354 | (9.9%) | 2237 | (5.1%) |
| **Dose Per Fractionation** | |  |  |  |  |  |  |  |  |
|  | 2.5Gy/Fx | 5193 | (54.6%) | 2029 | (21.3%) | 1240 | (13.0%) | 1056 | (11.1%) |
|  | 3Gy/Fx | 18375 | (60.0%) | 6001 | (19.6%) | 3746 | (12.2%) | 2502 | (8.2%) |
|  | 4Gy/Fx | 4977 | (69.1%) | 1259 | (17.5%) | 656 | (9.1%) | 309 | (4.3%) |
|  | 8Gy/Fx | 3286 | (91.6%) | 207 | (5.8%) | 67 | (1.9%) | 26 | (0.7%) |
| **Met Location** | |  |  |  |  |  |  |  |  |
|  | Spine | 17252 | (57.9%) | 5880 | (19.7%) | 3907 | (13.1%) | 2743 | (9.2%) |
|  | Shoulder | 1199 | (66.9%) | 318 | (17.7%) | 172 | (9.6%) | 103 | (5.7%) |
|  | Ribs | 1229 | (70.0%) | 309 | (17.6%) | 140 | (8.0%) | 78 | (4.4%) |
|  | Pelvic bones | 7029 | (68.4%) | 1766 | (17.2%) | 896 | (8.7%) | 580 | (5.6%) |
|  | Extremity, NOS | 2941 | (74.3%) | 616 | (15.6%) | 247 | (6.2%) | 153 | (3.9%) |
|  | Other | 2181 | (64.7%) | 607 | (18.0%) | 347 | (10.3%) | 236 | (7.0%) |

**Supplemental Table 6: Binary Logistic Regression of Randomized cohort with assumed 30Gy/10Fx course evaluating ≥25% PRLSRT**

| **Variables** (n=25301) | | **Binary Logistic Regression for ≥25% PRLSRT** | | | |
| --- | --- | --- | --- | --- | --- |
|  |  | Odds  Ratio | Lower  CI | Upper  CI | p-value |
| **Primary Tumor Site** | |  |  |  |  |
|  | Breast |  |  |  | <0.001 |
|  | Head and Neck | 2.549 | 1.664 | 3.902 | <0.001 |
|  | Upper GI | 4.373 | 3.733 | 5.123 | <0.001 |
|  | Lower GI | 2.689 | 2.124 | 3.403 | <0.001 |
|  | GI NOS | 5.63 | 3.827 | 8.282 | <0.001 |
|  | Lung (SC) | 2.87 | 2.385 | 3.453 | <0.001 |
|  | Lung (NSC) | 4.13 | 3.623 | 4.709 | <0.001 |
|  | Skin | 3.567 | 2.515 | 5.059 | <0.001 |
|  | Gynecologic | 3.263 | 2.337 | 4.554 | <0.001 |
|  | Genitourinary | 2.412 | 2.045 | 2.844 | <0.001 |
|  | Prostate | 0.838 | 0.686 | 1.023 | 0.082 |
|  | Endocrine | 1.341 | 0.807 | 2.229 | 0.258 |
| **Met Location** | |  |  |  |  |
|  | Ribs |  |  |  | <0.001 |
|  | Extremity, NOS | 1.201 | 0.953 | 1.513 | 0.122 |
|  | Shoulder | 1.399 | 1.076 | 1.817 | 0.012 |
|  | Pelvic bones | 1.473 | 1.2 | 1.81 | <0.001 |
|  | Spine | 2.217 | 1.824 | 2.695 | <0.001 |
|  | Other | 1.789 | 1.421 | 2.252 | <0.001 |
| **Age** | |  |  |  |  |
|  | Age ≥70 | 1.947 | 1.823 | 2.079 | <0.001 |
| **Met Organ Involvement** | |  |  |  |  |
|  | Bone Only |  |  |  | <0.001 |
|  | Bone w/Brain | 1.313 | 1.125 | 1.533 | 0.001 |
|  | Bone w/Liver | 1.734 | 1.573 | 1.911 | <0.001 |
|  | Bone w/Lung | 1.274 | 1.148 | 1.414 | <0.001 |
|  | Bone w/Other | 1.396 | 1.125 | 1.733 | 0.002 |
|  | Bone w/Multiple | 2.017 | 1.837 | 2.215 | <0.001 |
| **Charlson-Deyo Score** | |  |  |  |  |
|  | 0 |  |  |  | <0.001 |
|  | 1 | 1.301 | 1.203 | 1.407 | <0.001 |
|  | 2 | 1.559 | 1.385 | 1.755 | <0.001 |
|  | 3 or more | 2.168 | 1.853 | 2.536 | <0.001 |


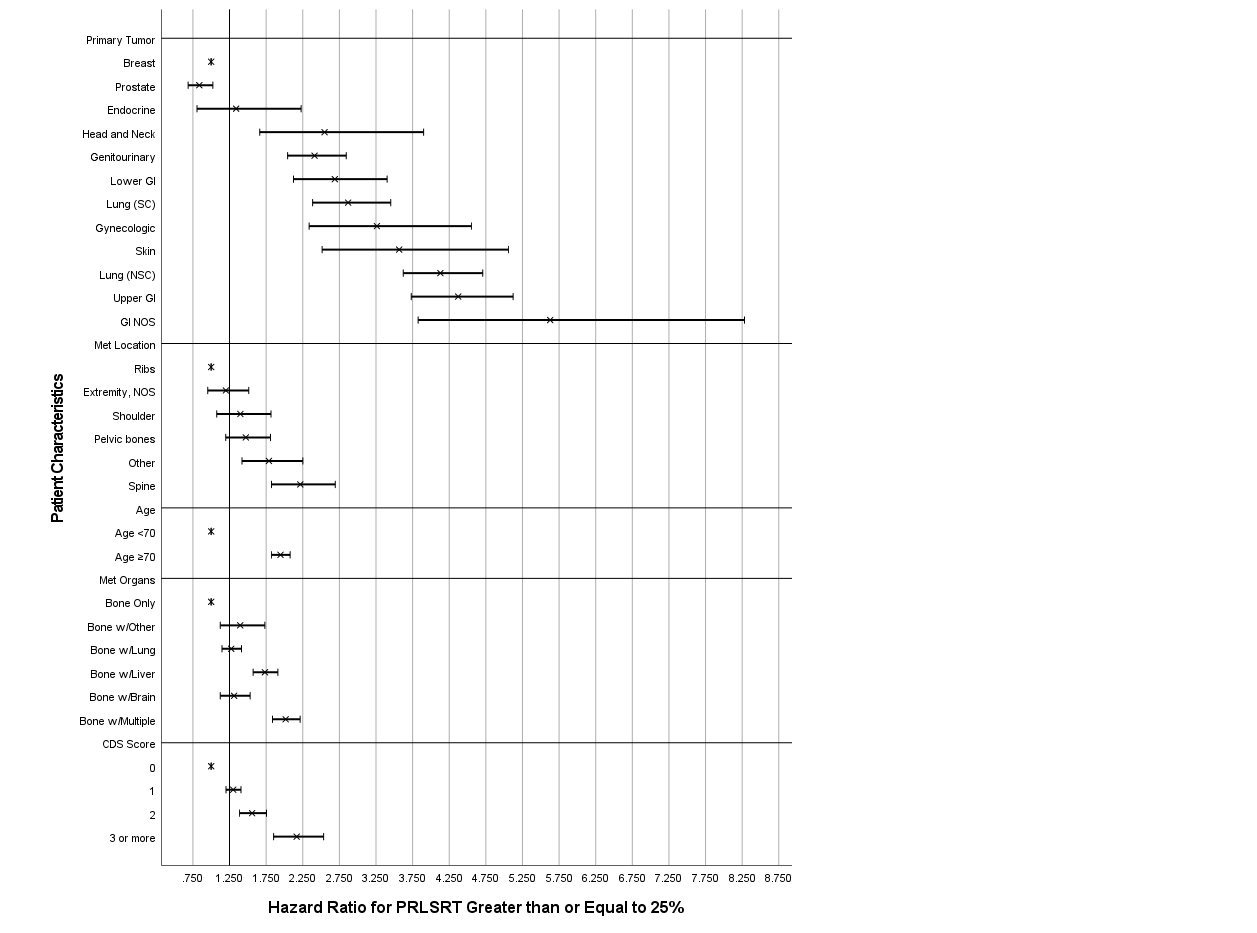

Supplement: Supplementary file 1 [file DataSheet1.docx]
